# Leu452His Mutation in Lipoprotein Lipase Gene Transfer Associated with Hypertriglyceridemia in Mice *in vivo*

**DOI:** 10.1371/journal.pone.0075462

**Published:** 2013-09-26

**Authors:** Kaiyue Sun, Wei Yang, Yanna Huang, Yizhen Wang, Lan Xiang, Jianhua Qi

**Affiliations:** 1 College of Pharmaceutical Sciences, Zhejiang University, Hangzhou, China; 2 Institute of Feed Sciences, Zhejiang University, Key Laboratory of Molecular Animal Nutrition, Ministry of Education, Key Laboratory of Feed and Animal Nutrition of Zhejiang Province, Hangzhou, China; The Chinese University of Hong Kong, Hong Kong

## Abstract

Mutated mouse lipoprotein lipase (LPL) containing a leucine (L) to histidine (H) substitution at position 452 was transferred into mouse liver by hydrodynamics-based gene delivery (HD). Mutated-LPL (MLPL) gene transfer significantly increased the concentrations of plasma MLPL and triglyceride (TG) but significantly decreased the activity of plasma LPL. Moreover, the gene transfer caused adiposis hepatica and significantly increased TG content in mouse liver. To understand the effects of MLPL gene transfer on energy metabolism, we investigated the expression of key functional genes related to energy metabolism in the liver, epididymal fat, and leg muscles. The mRNA contents of hormone-sensitive lipase (HSL), adipose triglyceride lipase (*ATGL*), fatty acid-binding protein (FABP), and uncoupling protein (*UCP*) were found to be significantly reduced. Furthermore, we investigated the mechanism by which MLPL gene transfer affected fat deposition in the liver, fat tissue, and muscle. The gene expression and protein levels of forkhead Box O3 (FOXO3), AMP-activated protein kinase (AMPK), and peroxisome proliferator-activated receptor-gamma coactivator 1 alpha (PGC-1α) were found to be remarkably decreased in the liver, fat and muscle. These results suggest that the Leu452His mutation caused LPL dysfunction and gene transfer of MLPL *in vivo* produced resistance to the AMPK/PGC-1α signaling pathway in mice.

## Introduction

Lipoprotein lipase (LPL), a 55 kDa glycoprotein, is a rate-limiting enzyme for the hydrolysis of triglyceride (TG)-rich lipoprotein. LPL is mainly expressed in adipose tissues, skeletal muscle tissues, and cardiac muscle tissues [1,2], and only its non-covalent dimer is active [3]. Active LPL is bound to the surface of endothelial cells and can be released into the blood by heparin. LPL hydrolyzes chylomicrons and very low-density lipoproteins to release free fatty acids (FFAs), produce remnant lipoproteins, and induce high-density lipoprotein formation [1]. FFAs are involved in energy metabolism or are stored as TGs [4]. Genetic defects in LPL are responsible for the reduction in TG-rich lipoprotein clearance, and mutations in the LPL gene play important roles in the development of hypertriglyceridemia in the general population [5–7]. To date, approximately 143 different mutations have been found in the human LPL gene, 90% of which occur in the coding regions and affect LPL functions through catalytic activity, dimerization, secretion, and heparin bonding [8].

Type 2 diabetes is correlated with obesity, coronary disease, atherosclerosis, and hypertension. Disorders in fatty acid metabolism are associated with development of these diseases. Given that LPL is a key enzyme in lipid metabolism, it is considered the main candidate gene for type 2 diabetes. Recent evidence has shown that plasma LPL has significant correlation with insulin sensitivity but negative correlation with insulin resistance and fasting insulin sensitive index [9,10]. In a study of Chinese population with type 2 diabetes and hypertriglyceridemia, 10 mutations were found in the LPL genes of diabetic patients. They contained four missense mutations (Ala71Thr, Val181Ile, Gly188Glu, and Glu242Lys), one nonsense mutation (Ser447Ter), and five silent mutations [6,11]. The four missense mutations, which occurred in the highly conservative amino acid sites in exons 3, 5, and 6, caused LPL dysfunction and hypertriglyceridemia. LPL dysfunction can increase the amount of FFAs in the body and further contribute to insulin resistance.

Many LPL transgenic models have been made to date. Overexpression of the human LPL remarkably ameliorates hypertriglyceridemia in transgenic Watanabe heritable hyperlipidemic rabbits. Increased LPL activity in male transgenic Watanabe heritable hyperlipidemic rabbits corrects hypercholesterolemia and reduces body fat accumulation [12]. In addition, systemic overexpression of LPL increases whole-body insulin sensitivity [13]. Furthermore, gene transfer of human LPL can ameliorate hyperlipidemias associated with apolipoprotein E and LDL receptor deficiencies in mice [14]. Notably, overexpression of human LPL in mouse skeletal muscle is associated with insulin resistance [15]. However, the effect of overexpression of mutated-LPL (MLPL) on energy metabolism within peripheral tissues (e.g., fat and muscle) is unknown.

In our previous studies, we investigated whether key functional genes related to energy metabolism, such as leptin and adiponectin, take part in lipid deposition and fibromuscular development in muscles by using overexpression of leptin or adiponectin gene. In the present study, to investigate whether LPL has the same effects on lipid deposition in muscles, we constructed pTarget/LPL vector and found a leucine (L) instead of a histidine (H) at position 452 of mouse LPL via sequence analysis. To identify whether this mutation and MLPL gene transfer affect the physiological function of LPL and the energy metabolism of peripheral tissues, respectively, we expressed the MLPL gene in the mouse liver by hydrodynamics-based gene delivery (HD). In this study, we report that the Leu452His mutation of LPL causes LPL dysfunction and inhibits energy expenditure of peripheral tissues (e.g., fat and muscle) via antagonistic effects after MLPL gene transfer.

## Materials and Methods

### Construction of plasmid DNA vector

Plasmid DNA-encoding mouse MLPL was constructed by inserting the cDNA clone of LPL from the epididymal fat of Institute of Cancer Research (ICR) mouse into the pTarget vector (Promega, Wisconsin, USA). Total RNA was extracted using an RNA extraction kit (Beijing ComWin Biotechnology, Beijing, China) to prepare the cDNA clone of LPL. RT-PCR was performed to retrieve the full-length mouse LPL cDNA (GenBank Accession No. NM_16956 LPL) using a cDNA synthesis kit (Beijing ComWin Biotechnology, Beijing, China). The mouse LPL specific primers used for PCR reaction were as follows: sense, 5′-ATG GAG AGC AAA GCC CTG-3′; and antisense, 5′-TCA GCC AGA CTT CTT CAG AG-3′. The PCR conditions were as follows: pre-denaturation at 93°C for 3 min; 30 cycles of 94°C for 50 s, 56°C for 45 s, and 72°C for 30 s; and a complete cycle after 72°C for 10 min. The amplified products were analyzed by electrophoresis, and the specific 1425 bp band of the amplified cDNA fragment was confirmed. All cDNA fragments were subsequently gel-purified using a DNA purification kit (Beijing ComWin Biotechnology, Beijing, China). The cloned LPL cDNA was inserted into the pTarget vector of the pTarget^TM^ mammalian expression vector system (Promega, Wisconsin, USA). The reaction reagent was transformed into TOP 10 competent cells, which were smeared on ampicillin agar plates and incubated overnight at 37°C. The clones that formed on the agar plates were individually amplified, and plasmid DNA was extracted with a mini DNA purification kit (Beijing ComWin Biotechnology, Beijing, China). It was cut with *EcoR*I (Takara, Otsu, Japan), and the sizes of the inserted fragment and pTarget vector were checked by 1% gel electrophoresis. Individual LPL clones were subsequently obtained from each pTarget/LPL and then sequenced to confirm the fidelity of mouse LPL. Large amplification of the plasmid DNA was prepared using the Endo Free Plasmid Maxi Kit (Qiagen, Nordrhein Westfalen, Germany) to remove bacterial endotoxins.

### Experimental Animals

Five-week-old male ICR strain mice (n=30) were used as experimental animals. They were maintained in a clean room at 23°C ±1°C with a 12:12 light–dark cycle and fed with a commercial diet (SLRC, Shanghai, China). All experiments were carried out with strict adherence to the Guide for the Care and Use of Laboratory Animals of the National Institutes of Health. The protocol was approved by the Committee on the Ethics of Animal Experiments of Zhejiang University (Permit Number: Zju2010101034).

### Hydrodynamics-based gene delivery

MLPL gene transfer was performed as described in a previous study [16]. The mice were maintained during the subsequent week after HD to record food intake and changes in body weight. Blood was extracted for analysis of plasma glucose, TG, and MLPL+LPL concentrations and LPL activity at designated time points after injection of 1% heparin for 10 min during the experiment. On day 1 after HD and at the end of the experiment, the treated mice were sacrificed by neck dislocation. The liver, epididymal white fat, *gastrocnemius*, and *extensor digitorum longus* were removed quickly and weighed.

### ELISA and LPL activity assays

On days 1 and 7 after MLPL gene transfer, plasma samples were obtained by centrifuging the blood at 12,000 rpm for 15 min at room temperature (RT) and storing at -20°C until analysis. The plasma samples were assayed for MLPL+LPL concentration using a commercially available mouse LPL ELISA Kit (Xitang, Shanghai, China) according to the manufacturer’s protocol. The plasma LPL activity of the blood samples was measured using an LPL Activity Assay Kit (Bioengineering Institute of Nanjing Jiancheng Company, Nanjing, China) according to the manufacturer’s protocol.

### Plasma glucose and TG assay

The glucose and TG concentrations in blood serum samples on days 1 and 7 after MLPL gene transfer were measured with a plasma glucose assay kit (Roche, Basel, Swiss) and mouse TG ELISA kit (Roche, Basel, Swiss), respectively, following the manufacturer’s protocol.

### Liver TG measurement

Approximately 200 mg of each liver sample obtained on day 7 after the gene transfer was homogenized in methanol at 1:2 ratios by volume. Six volumes of chloroform was added into the homogenate and extracted for 12 hours at RT. Subsequently, TG content was measured with a TG assay kit (Beijing Beihuakangtai Company, Beijing, China) according to the manufacturer’s protocols.

### Real-Time PCR

Approximately 50 mg samples of the liver, *gastrocnemius*, *extensor digitorum longus*, and epididymal white fat were individually obtained. Total RNA was extracted using the Trizol Reagent (Invitrogen, California, USA), and RNA content was determined using a spectrophotometer. Transcription was performed using 2.5 µg of total RNA, Oligo (dT)_20_ (Invitrogen, California, USA) as primers, and reverse transcriptase (Fermentas, Shenzhen, China). The transcript levels were quantified by real-time PCR (AB SCIEX, Massachusetts, USA) and SYBR Premix EX Taq^TM^ (Takara, Otsu, Japan). The mouse *HSL*, *ATGL*, *FABP3*, *FABP4*, *UCP2*, *UCP3*, *LPL*, *AMPK*, *SIRT1*, *FOXO3a*, *PGC-1α*, and *18S* RNA primers used for the PCR were as follows: for *HSL*: sense, 5′-TGA GAT GGT AAC TGT GAG CC-3′, antisense, 5′-ACT GAG ATT GAG GTG CTG TC-3′; for *ATGL*: sense, 5′-AAC ACC AGC ATC CAG TTC AA-3′, antisense, 5′-GGT TCA GTA GGC CAT TCC TC-3′; for *FABP3*: sense, 5′-TCA GCT GGG AAT AGA GTT CGA C-3′, antisense, 5′-TAG TTA GTG TTG TCT CCT GCC C-3′; for *FABP4*: sense, 5′-GAT GAA ATC ACC GCA GAC GAC A-3′, antisense, 5′-ATT GTG GTC GAC TTT CCA TCC C-3′; for *UCP2*: sense, 5′-TCC CCT GTT GAT GTG GTC AA-3′, antisense, 5′-CAG TGA CCT GCG CTG TGG TA-3′; for *UCP3*: sense, 5′- CCT ACG ACA TCA TCA AGG AGA AGT T-3′, and antisense, 5′-TCC AAA GGC AGA GAC AAA GTG A-3′; for *LPL*: sense, 5′-CTG GGC TAT GAG ATC AAC AAG GT-3′, and antisense, 5′-AGG GCA TCT GAG AGC GAG TCT-3′; for *AMPK*: sense, 5′- TTG ACG ATG AGG CTG TGA AG-3′, antisense, 5′- ATA AGC CAC TGC AAG CTG GT-3′; for *SIRT1*: sense, 5′-CTG CCA CAA GAA CTA GAG GAT AAG A-3′, antisense, 5′-TGG CAA AGG AGC AGA TTA GTA GG-3′; for *PGC-1*α sense, 5′-CCG AGA ATT CAT GGA CAA T-3′, and antisense, 5′-GTG TGA GGA GGG TCA TCG TT -3′; for *FOXO3a*: sense, 5′-AGT CTC CCA TGC AGA CCA TC-3′, and antisense, 5′-GAG TCC GAA GTG AGC AGG TC-3′; for *18S RNA*: sense, 5′- TAA CCC GTT GAA CCC CAT T-3′, and antisense, 5′- CCA TCC AAT CGG TAG TAG CG-3′. Using Takara SYBR Premix Ex Taq, the cDNA was amplified under the following conditions: 95°C for 30 s, followed by 40 cycles for 5 s at 95°C, and 34 s at 60°C. All results were normalized to *18S* RNA levels, and relative mRNA transcript levels were calculated using the ΔΔCt formula. All samples were run in triplicate and the average values were calculated.

### Western blot analysis

Liver and muscle samples (200 mg and 50 mg each) obtained on days 1 and 7 after the gene transfer, respectively, were homogenized in lysis buffer supplemented with protease inhibitors. Soluble proteins were recovered after centrifugation at 12,000 rpm for 20 min at 4°C. Subsequently, protein concentration was spectrophotometrically measured using a Bio-Rad protein assay kit (Bio-Rad Laboratories, California, USA) at 595 nm. A sample corresponding to 100 micrograms protein was removed, added to a new tube, and then incubated at 100°C for 20 min. Then, 11% SDS-PAGE was run at 200 V for 45 min. The proteins were transferred to a polyvinylidene difluoride membrane and then blocked with 5% non-fat dry milk in 0.05% Tris-buffered saline-Tween 20 for 1 hour at RT. Blots were incubated with anti-FOXO3 (R&D Systems, Shanghai, China), anti-AMPK, anti-phospho-AMPK (phospho-Ser487) (Signalway Antibody, Nanjing, China), anti-PGC-1α (Abcam, Hong Kong, China), and β-actin (Abmart, Shanghai, China) antibodies in locking buffer overnight at 4°C. After washing five times with 0.5% TBS-Tween, the membranes were incubated with horseradish peroxidase-conjugated secondary antibody (Beijing ComWin Biotechnology, Beijing, China) for 1 hour at RT. Enhanced chemiluminescent reaction (GE HealthCare, New Jersey, USA) was used to develop the bands, which were analyzed by density analysis using the ImageJ software (National Institute of Health, Maryland, USA).

### Determination of intracellular ATP level of liver, epididymal white fat, *gastrocnemius* and *extensor deigitorum longus* after gene transfer

The ATP level of tissues was measured by using an ATP assay kit (Beyotime Institute of Biotechonogy, Haimen, China) and Luminometer 20/20^n^ (Turner Biosystems, California, USA) following the respective manufacturer’s instructions. Briefly, approximately 200 mg of each liver sample obtained on day 1 and day 7 after the gene transfer was homogenized and lysed in ATP assay lysis buffer at 1:5 ratio by volume. At the same time, 50 mg of epididymal white fat, *gastrocnemius* and *extensor deigitorum longus* of each sample was treated by the same method. The supernatant was removed after centrifugation at 12,000 rpm for 10 min at 4°C. Then, 100 µl ATP assay buffer was added to a new tube, followed by addition of supernatant to a final volume of 120 μl/tube. The relative light unit was read using a luminometer. The ATP content of each sample was calculated based on a standard curve. ATP level was normalized to the total protein concentrations of tissue lysates determined using the Bio-Rad protein assay kit.

### Determination of mutated-LPL structure change

Native PAGE was performed as previously described [17] to check whether the plasma MLPL in the treatment groups underwent structural changes. Plasma samples of the control and treatment groups (15 µg) without denaturation were loaded in 9% acrylamide gel [4.4 mL H_2_O, 3 mL 30% acrylamide, 2.5 mL 1.5 M/L Tris (pH 8.8), 0.1 mL 10% ammonium persulfate, and 0.005 mL N, N, N’, N’-Tetra-methylethylenediamine]. After gel electrophoresis was run at 20 mA for 1.5 hours, the gel was stained with Coomassie brilliant blue reagent (Beijing ComWin Biotechnology, Beijing, China) according to the manufacturer’s protocol. Photographs of the gel were taken, and the dimer or monomer of MLPL was determined based on difference in molecular weight. Furthermore, western blot was conducted to detect MLPL using a human LPL antibody (Santa Cruz, California, USA).

### Statistical Analysis

Data are expressed as mean ± standard error of the mean (SEM). Significant differences between groups in all experiments were determined by ANOVA using SPSS 15.0. *P*-values less than 0.05 were considered to indicate statistical significance.

## Results

### Construction of the expression plasmid and amino acid sequence analysis

**Figure 1:**
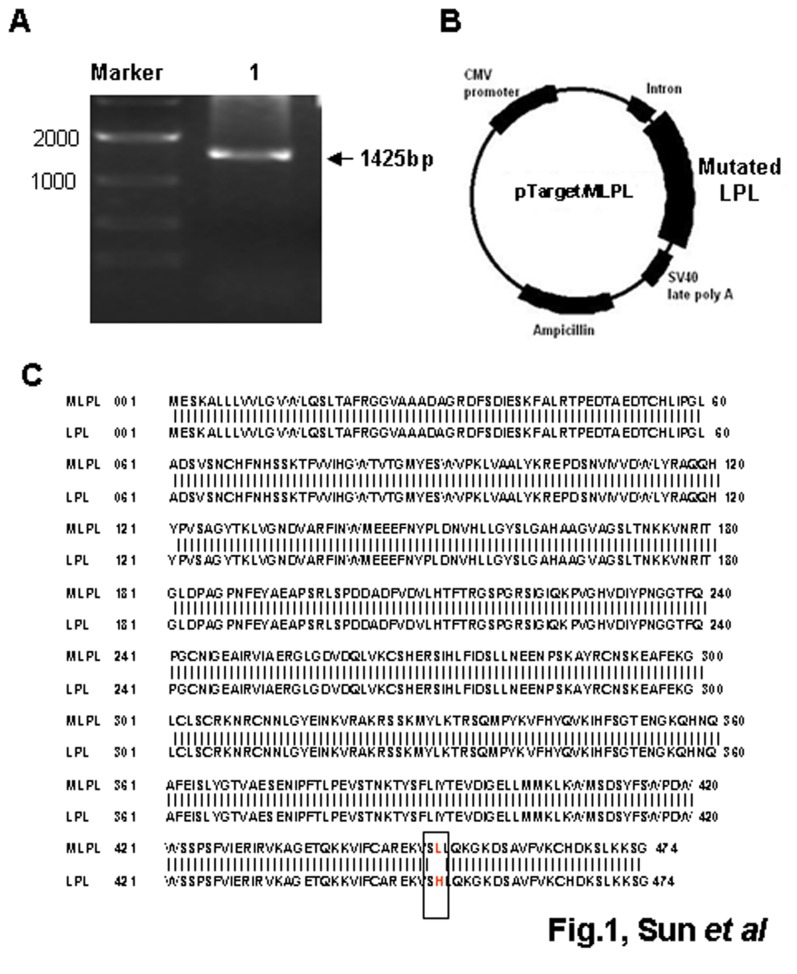
Construction of pTarget/MLPL expression vector. (A) MLPL cDNA fragment obtained by PCR amplification. Lanes: M, 2000 bp marker; 1, MLPL cDNA fragment. (B) Mapping of pTarget/MLPL. (C) Sequence analysis of MLPL amino acid in plasmid pTarget/MLPL.

To construct the pTarget/MLPL expression vector, total RNA was extracted from the epididymal fat of ICR mice, and cDNA was obtained by RT-PCR. The specific 1425 bp fragment of MLPL cDNA was verified using 1% gel electrophoresis (Figure 1A). The cDNA was then inserted in the pTarget vector of the pTarget^TM^ mammalian expression vector system, resulting in the construction of pTarget/MLPL (Figure 1B). Sequence analysis was performed on the inserted fragment of plasmid DNA, yielding an amino acid sequence containing one mutation at position 452 compared with that of the GenBank Accession No. NM 16956 LPL (Figure 1C). Thus, the pTarget/MLPL expression vector contained a mutation at position 452 (an L instead of an H).

### Detection of MLPL mRNA in the liver and plasma MLPL+LPL concentration and LPL activity

**Figure 2:**
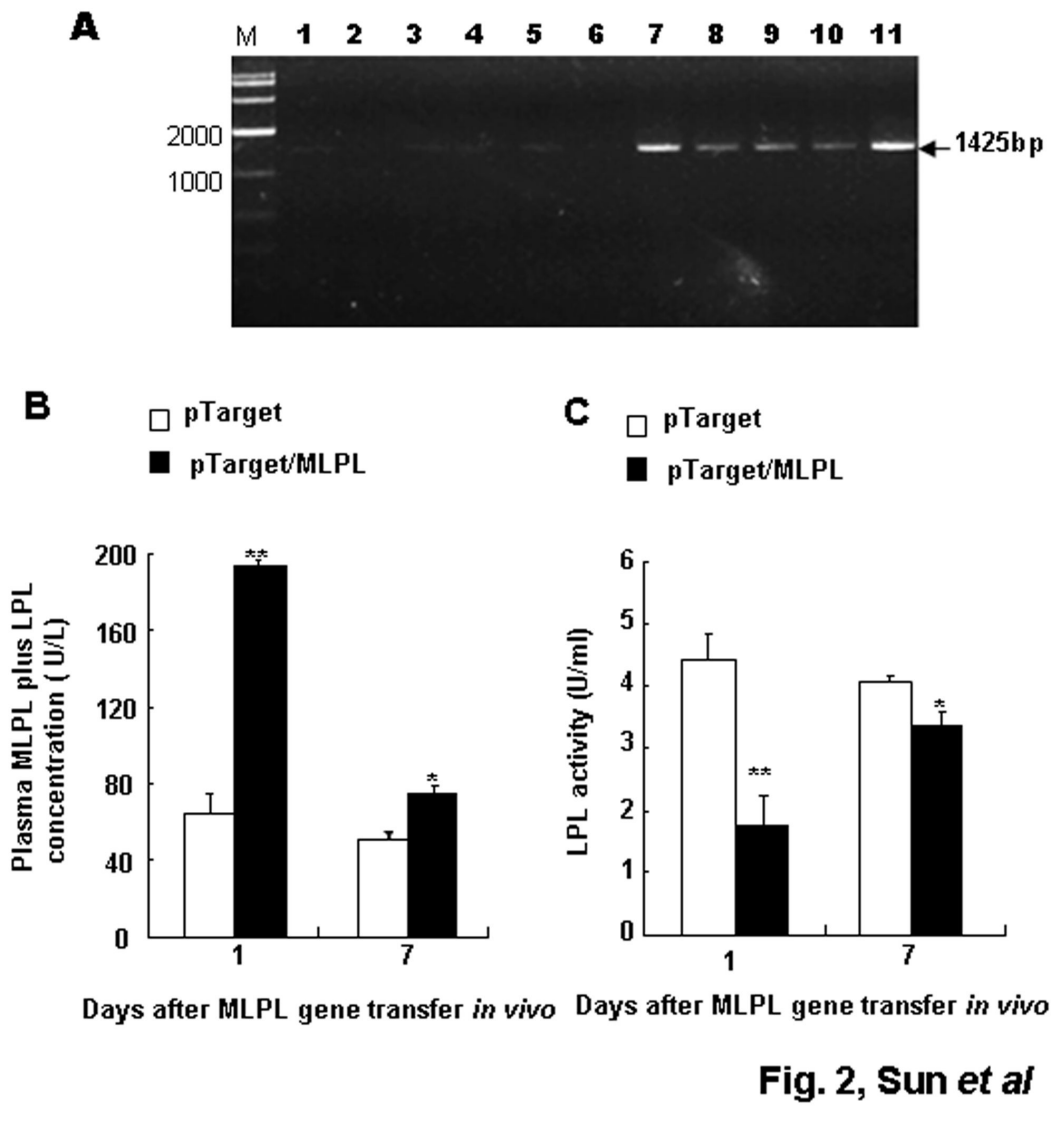
MLPL gene expression in the liver and change in plasma MLPL+LPL concentration and LPL activity. Analysis of pTarget/MLPL transcript in the liver, changes in plasma MLPL +LPL concentration and LPL activity. (A) RT-PCR-amplified MLPL mRNA in the mouse liver to which pTarget/MLPL was transferred *in* *vivo* by HD gene delivery at a dose of 15 micrograms per animal. Lanes: M, 2000 bp marker; 1–6, control livers from six mice; 7–11, pTarget/MLPL transferred livers from five mice. Change in the concentration (B) and activity (C) of plasma MLPL+LPL and LPL after gene transfer *in* *vivo*. Each value represents mean ± SEM of five mice. * and ** indicate significant difference relative to the control at the same time point at p<0.05 and p<0.01, respectively.

Results of RT-PCR from the HD-treated liver on day 1 after the gene transfer are shown in Figure 2A. The observed specific bands for MLPL mRNA were 1425 bp in size. This result suggests that MLPL mRNA was adequately produced in the liver after HD gene transfer *in vivo*. The MLPL+LPL concentration in the plasma after the gene transfer is presented in Figure 2B. On day 1 post-transfection, a remarkable increase was detected compared with the control group on days 1 and 7 (p<0.01 and p<0.05, respectively), although MLPL+LPL concentration quickly decreased on day 7. However, the activity of plasma LPL on days 1 and 7 was significantly reduced compared with that in the control groups (Figure 2C) (p<0.01 and p<0.05). These variations imply that pTarget/MLPL was successfully transferred and that MLPL was produced in the liver. Furthermore, MLPL was secreted into the blood, and the Leu452His mutation in LPL caused LPL dysfunction.

### Changes in plasma glucose and TG after the gene transfer *in vivo*

**Figure 3:**
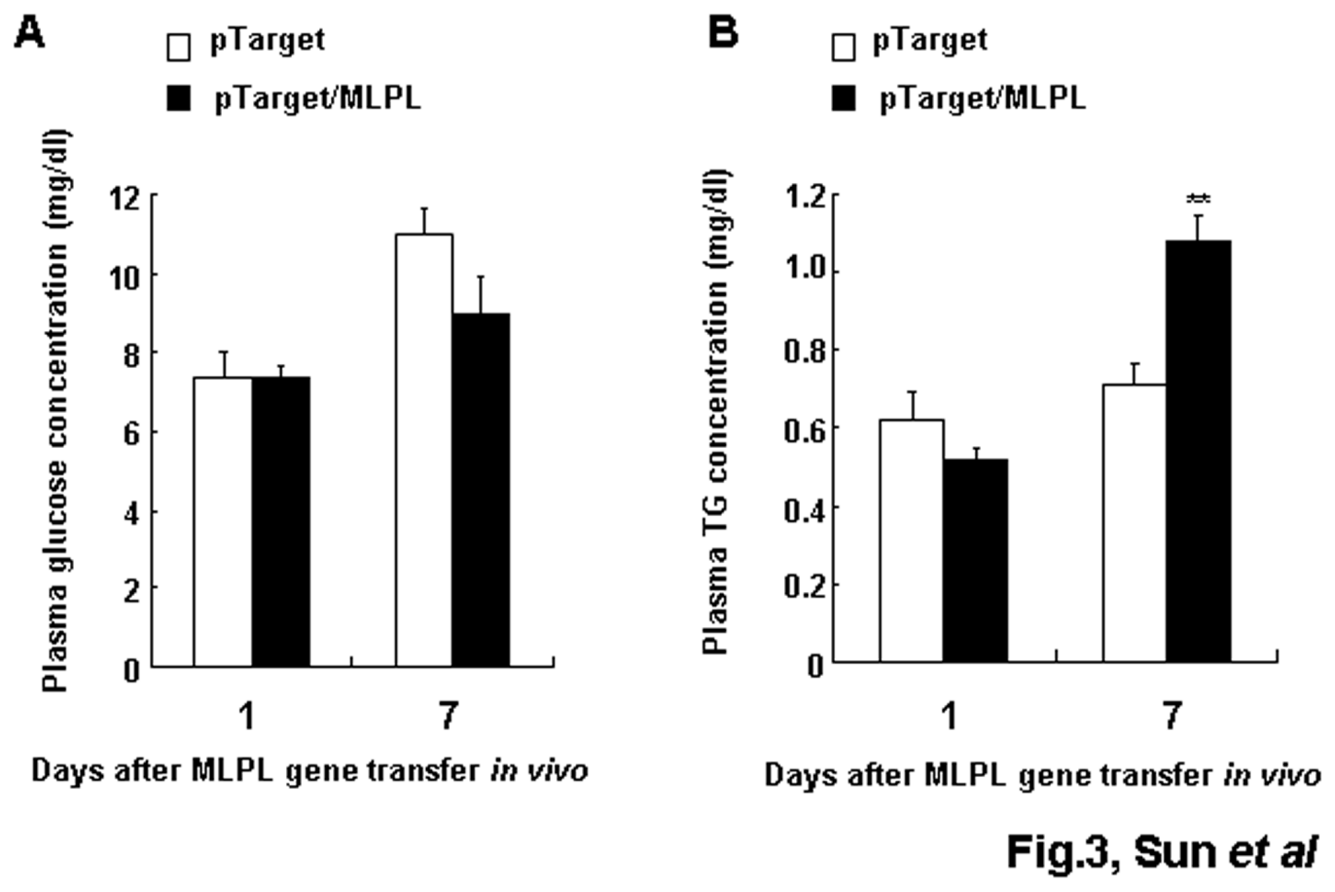
Effects of MLPL gene transfer on plasma glucose and TG. Variations in plasma glucose (A) and TG (B) after MLPL gene transfer *in* *vivo*. Each value represents mean ± SEM of five mice. ** indicates significant difference relative to the control at the same time point at p<0.01.

The concentrations of plasma glucose and TG on days 1 and 7 after the gene transfer are shown in Figure 3A and 3B. On day 7 after the gene transfer, the concentration of plasma TG remarkably increased (p<0.01). These results suggest that the MLPL produced in the liver after the gene transfer destroyed the physiological function of LPL and produced antagonistic effects against LPL by competitive inhibition.

### Changes in the liver and its TG content after the gene transfer *in vivo*

**Figure 4:**
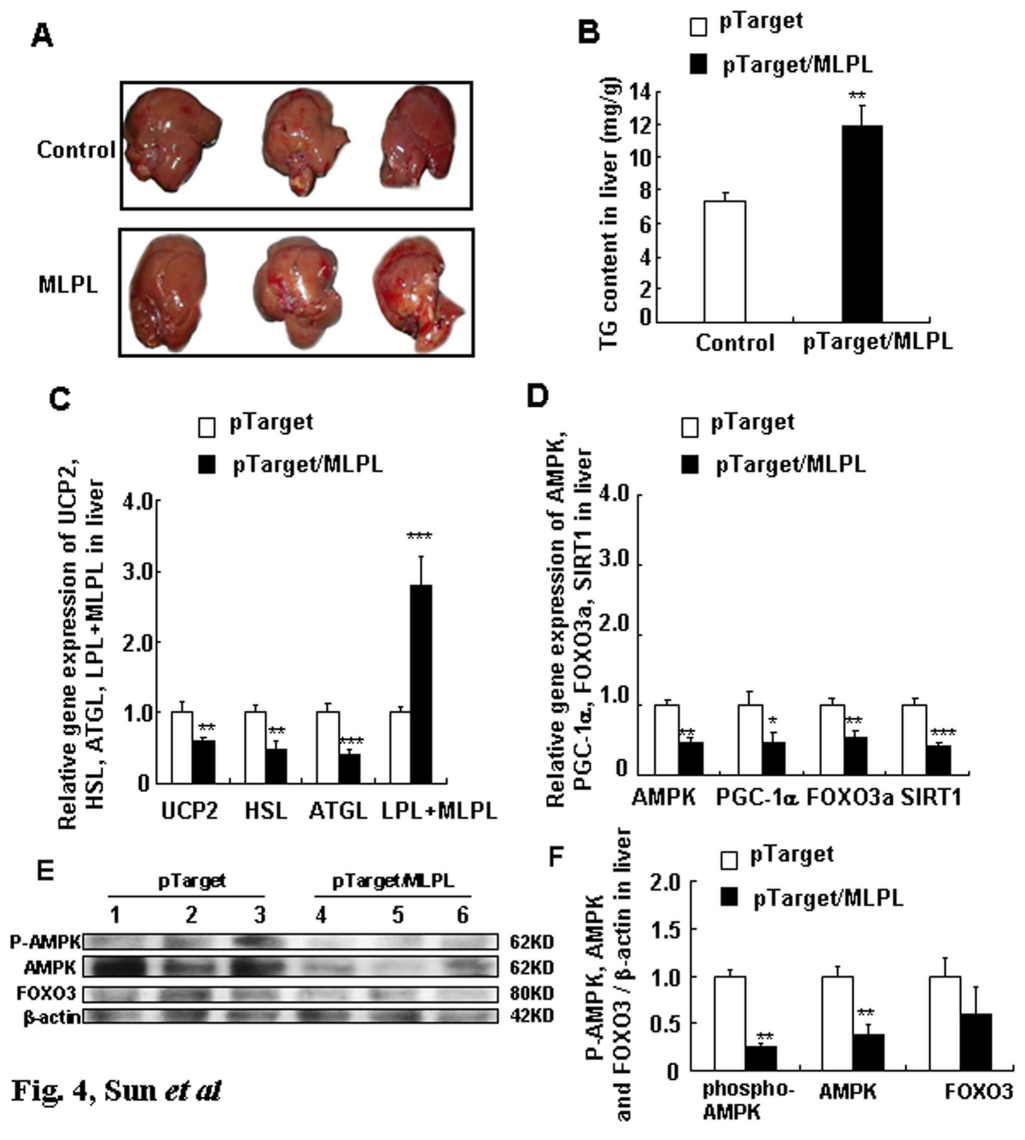
Effects of MLPL gene transfer on livers in mice *in vivo*. Images of the liver (A), variations in TG content (B), gene expression of key functional genes related to energy metabolism (C), and transcription factors (D). Changes in the protein level of transcription factors in the liver (E and F) after MLPL gene transfer *in* *vivo*. Each value represents mean ± SEM of five mice. *, ** and *** indicate significant difference relative to control at the same time point at p<0.05, p<0.01 and p<0.001, respectively.

Image of the liver and its TG content on day 7 after the gene transfer are presented in Figure 4A and 4B. Remarkable hepatic adipose infiltration was observed. Furthermore, the concentration of TG in the liver on day 7 after the gene transfer increased significantly (p<0.01). These results further indicate that MLPL may inhibit the effects of endogenous LPL.

### Effect of MLPL gene transfer on gene expression-related energy metabolism in the liver, epididymal white fat, and leg muscles

**Figure 5:**
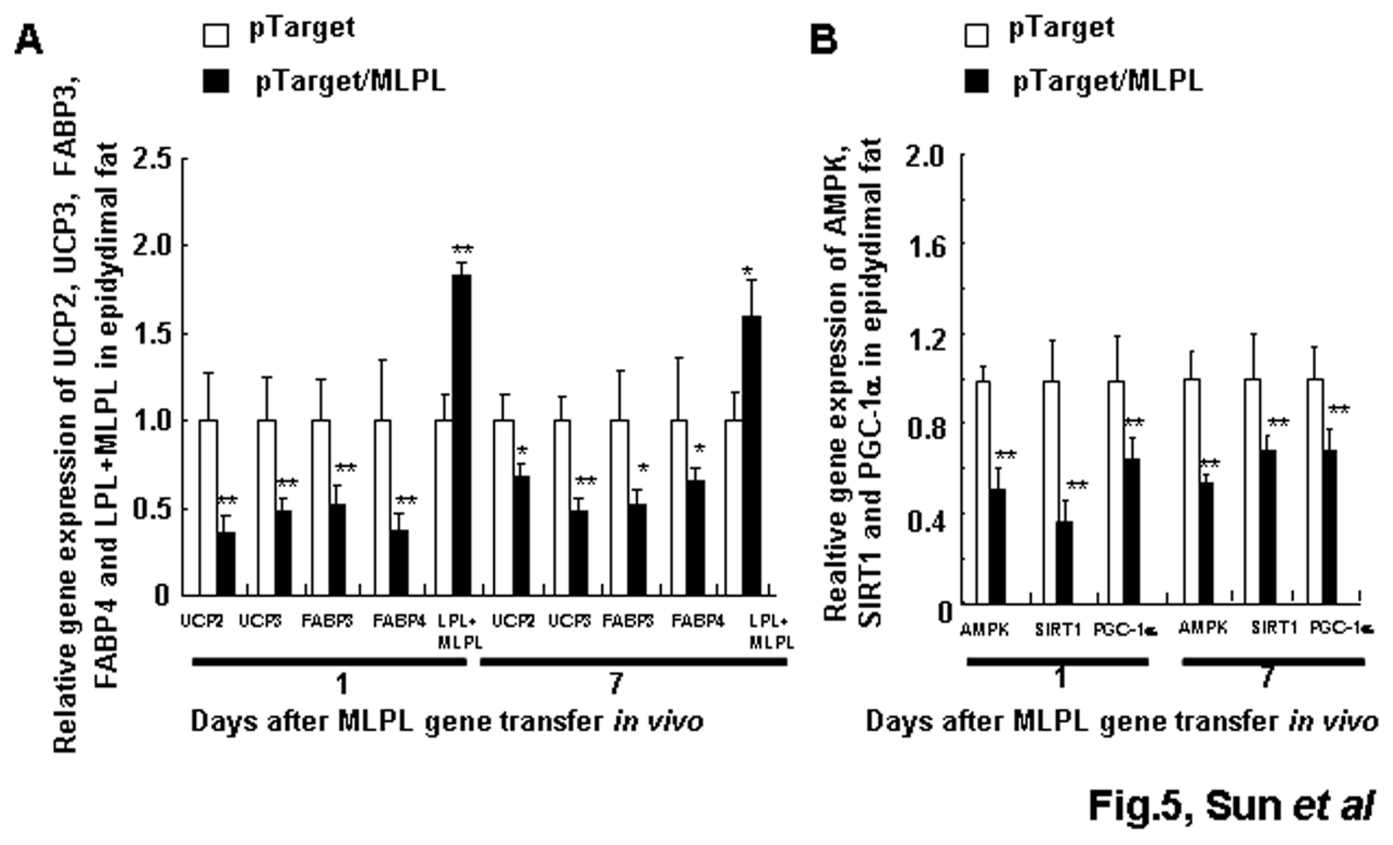
Effects of MLPL gene transfer on energy metabolism of fat tissue. Variations in the expression of key functional genes related to energy metabolism (A) and transcription factors (B) in epididymal white fat tissue after gene transfer *in* *vivo*. Each value represents mean ± SEM of five mice. * and ** indicates significant difference relative to the control at the same time point at p<0.05 and p<0.01, respectively.

**Figure 6:**
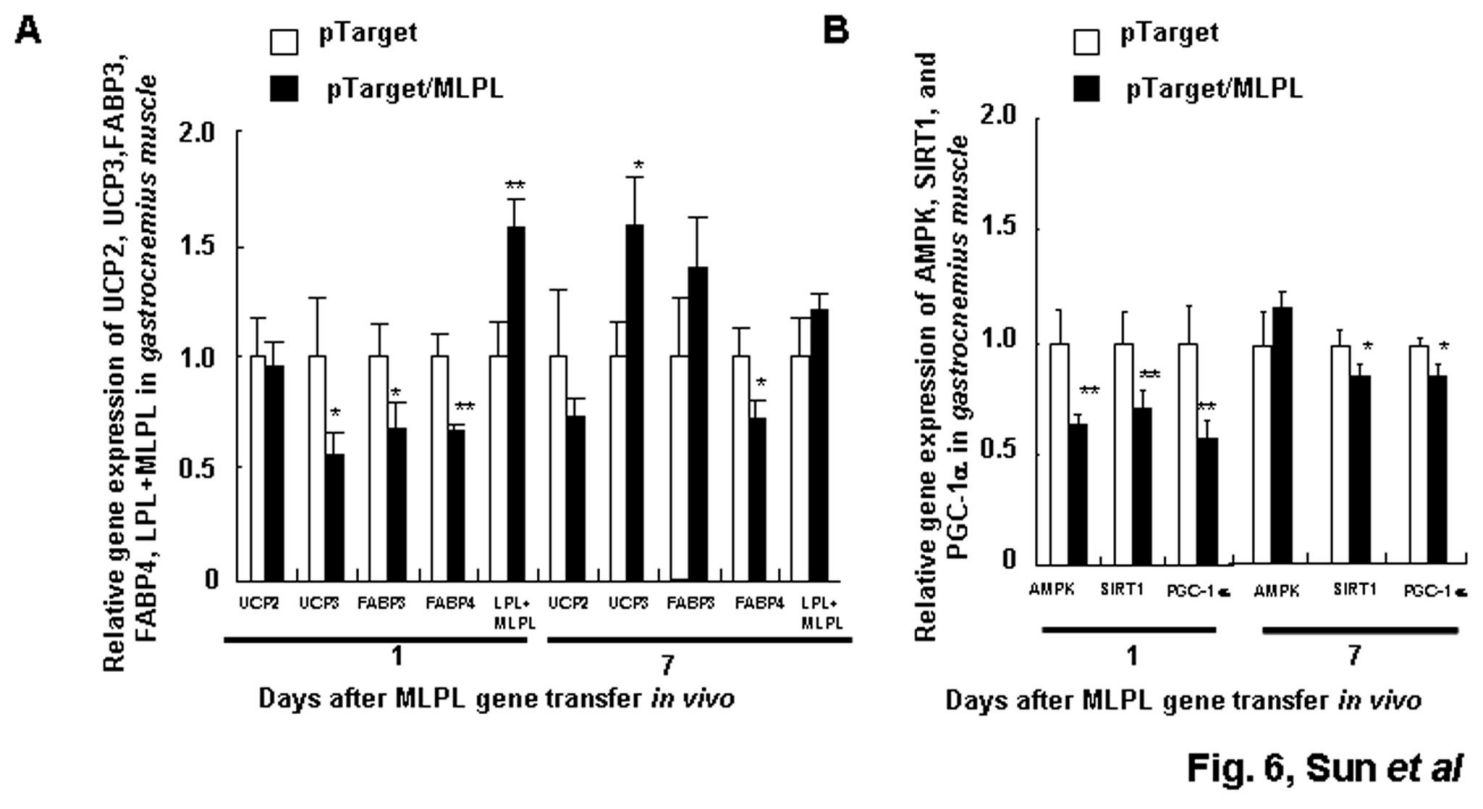
Effects of MLPL gene transfer on energy metabolism of *gastrocnemius*. Variations in expression of key functional genes related to energy metabolism (A) and transcription factors (B) in *gastrocnemius* after MLPL gene transfer *in* *vivo* by HD at a dose of 15 µg per animal. Each value represents mean ± SEM of five mice. * and ** indicate significant difference relative to the control at the same time point at p<0.05 and p<0.01, respectively.

**Figure 7:**
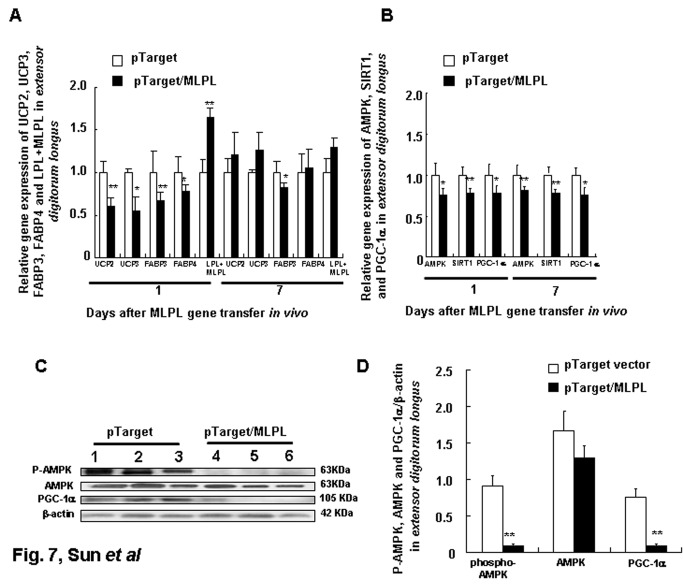
Effects of MLPL gene transfer on energy metabolism of *extensor digitorum longus*. Variations in expression of key functional genes related to energy metabolism (A) and transcription factors (B), AMPK, phospho-AMPK, and PGC-1α protein levels in *extensor* *digitorum* *longus* (C and D) muscle after MLPL gene transfer *in* *vivo* by HD at a dose of 15µg per animal. Each value represents mean + SEM of five or three mice. * and ** indicate significant difference relative to the control at the same time point at p<0.05 and p<0.01, respectively.

Figure 4C shows the gene expression profiles of *UCP2*, *HSL*, *ATGL*, and MLPL+*LPL* in the liver on day 1 after *MLPL* gene transfer. On day 1, *MLPL* gene transfer decreased the mRNA amounts of *UCP2*, *HSL*, and *ATGL* in the liver by 41.33%, 53.50%, and 58.44% (p<0.01, p<0.01, p<0.001), respectively, and increased the mRNA amount of *LPL+MLPL* in the liver by 179.35% (p<0.001). Figures 5A, 6A, and 7A display the expression levels of *UCP2*, *UCP3*, *FABP3*, *FABP4*, and *LPL+MLPL* in the epididymal white fat, *gastrocnemius*, and *extensor digitorum longus* on days 1 and 7 after MLPL gene transfer, respectively. Compared with the control groups, MLPL gene transfer reduced the mRNA amounts of *UCP2*, *UCP3*, *FABP3*, and *FABP4* in the epididymal white fat to 35.40%, 48.38%, 52.07%, and 37.58%, respectively, on day 1 (p<0.01 for all). Furthermore, they decreased to 67.75%, 48.38%, 52.07%, and 65.49% (p<0.05, p<0.01, p<0.05, p<0.05), respectively, on day 7. On day 1 after gene transfer, they were reduced to 95.82%, 55.62%, 67.53%, and 66.69% (p<0.01, p<0.05, and p<0.01), respectively, in the *gastrocnemius* and to 60.63%, 55.26%, 67.22%, and 77.82% (p<0.01, p<0.05, p<0.01, and p<0.05), respectively, in the *extensor digitorum longus* compared with those in the control groups. On day 7 after the gene transfer, the mRNA contents of *UCP3* and *FABP3* rebounded to 158.25% (p<0.05) and 139.50%, and the mRNA amounts of *UCP2* and *FABP4* were still reduced to 73.18% and 72.90% (p<0.05), respectively, in the *gastrocnemius*. At the same time, the mRNA amounts of *FABP3* still decreased to 81.61% (p<0.05), and the mRNA amounts of *UCP2*, *UCP3*, and *FABP4* were rebounded to 120.89%, 126.00%, and 105.15%, respectively, in the *extensor digitorum longus*. However, the mRNA amount of *LPL+MLPL* in the epididymal white fat, *gastrocnemius*, and *extensor digitorum longus* increased to 183.09%, 164.86%, and 157.25% (p<0.01, p<0.01, p<0.01), respectively, on day 1 and to 159.50% (p<0.05), 129.22%, and 120.56%, respectively, on day 7. These results reveal that MLPL gene transfer regulated fat deposition in the four samples by regulating the three aforementioned key genes related to energy metabolism.

Effect of MLPL gene transfer on AMPK, SIRT1, FOXO3a, and PGC-1α gene expressions, protein level, and AMPK phosphorylation in the liver, fat, *gastrocnemius muscle*, and *extensor digitorum longus*

The gene expression levels of *AMPK*, *SIRT1*, *PGC-1*α, and *FOXO3a* in the liver on day 1 after MLPL gene transfer are displayed in Figure 4D. On day 1 after MLPL gene transfer, the expression levels of *AMPK*, *PGC-1α*, *FOXO3a*, and *SIRT1* in the liver decreased by 53.45%, 51.51%, 46.21%, and 57.43%, respectively (p<0.01, p<0.05, p<0.01, and p<0.001). The gene expression levels of *AMPK*, *SIRT1*, and *PGC-1α* in epididymal white fat, *gastrocnemius*, and *extensor digitorum longus* on days 1 and 7 after MLPL gene transfer are presented in Figures 5B, 6B, and 7B. The mRNA contents of *AMPK*, *SIRT1*, and *PGC-1α* in the epididymal white fat decreased by 50.62%, 62.48%, and 43.22% (p<0.01, p<0.01, and p<0.01), respectively, on day 1 and to 55.06%, 75.56%, and 76.78% (p<0.01, p<0.01, and p<0.01), respectively, on day 7. The mRNA contents of *AMPK*, *SIRT1*, and *PGC-1α* mRNA in the *gastrocnemius* decreased to 72.91%, 75.33%, and 62.56% (p<0.01, p<0.01, and p<0.01), respectively, on day 1 and to 118.5%, 77.89%, and 77.69% (p<0.05 and p<0.05), respectively, on day 7. They were also decreased to 74.77%, 77.89%, and 77.69% (p<0.05, p<0.01, p<0.05) on day 1 and to 79.32%, 75.33%, and 72.97% (p<0.01, p<0.01, and p<0.05) on day 7, respectively, in the *extensor digitorum longus*. The protein levels of AMPK, phospho-AMPK, FOXO3, and PGC-1α in the liver and in the *extensor digitorum* are presented in Figure 4E and 4F, and in Figure 7C and 7D, respectively. The phospho-AMPK and AMPK proteins were obviously decreased in the liver (p<0.01, p<0.01). The PGC-1α and phospho-AMPK proteins were significantly reduced in the *extensor digitorum* (p<0.01, p<0.01). These results indicate that MLPL gene transfer might be reduction of energy expenditure by inhibiting expression of the transcription factors *AMPK*, *SIRT1*, and *PGC-1α* in the liver, epididymal white fat and leg muscles.

### Effects of MLPL gene transfer on ATP level of liver, epididymal white fat, *gastocnemius* and extensor *deigitorum longus*

**Figure 8:**
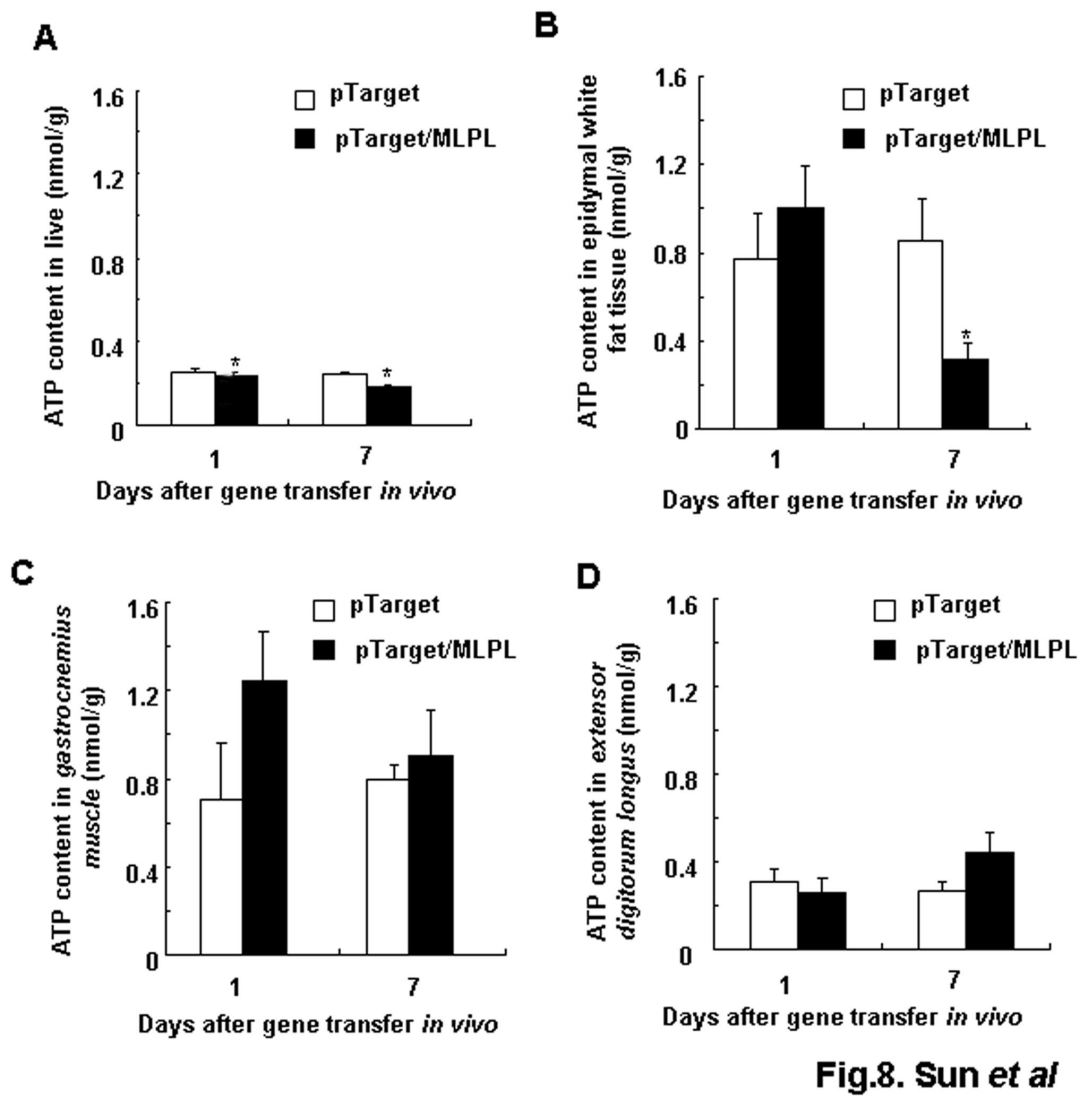
Effects of MLPL gene transfer on ATP level of liver, fat and muscles. Change in ATP level in the liver (A), epididymal white fat (B), *gastrocnemius* (C) and *extensor* *digitorum* *longus* (D). MLPL gene transferred liver, fat and leg muscle tissues from four mice. Each value represents mean + SEM of four mice. * indicates significant difference relative to the control at p<0.05.

To investigate the effects of MLPL expression on the level of ATP, we transferred the MLPL gene into mice *in vivo*, and measured ATP levels in the liver, epididymal white fat and leg muscles. As shown in Figure 8A to 8D, MLPL gene transfer significantly decreased the level of ATP in the liver only on day 1 and day 7 (p<0.05, p<0.05, respectively). The ATP level in fat tissues was also lowered at day 7 after gene transfer (p<0.05). However, the ATP level in leg muscles did not undergo significant change. This result suggests that the effects of MLPL on ATP level can vary depending on the type of tissue.

### Leu452His mutation of LPL did not affect the plasma bioactive dimerization of LPL after gene transfer *in vivo*

**Figure 9:**
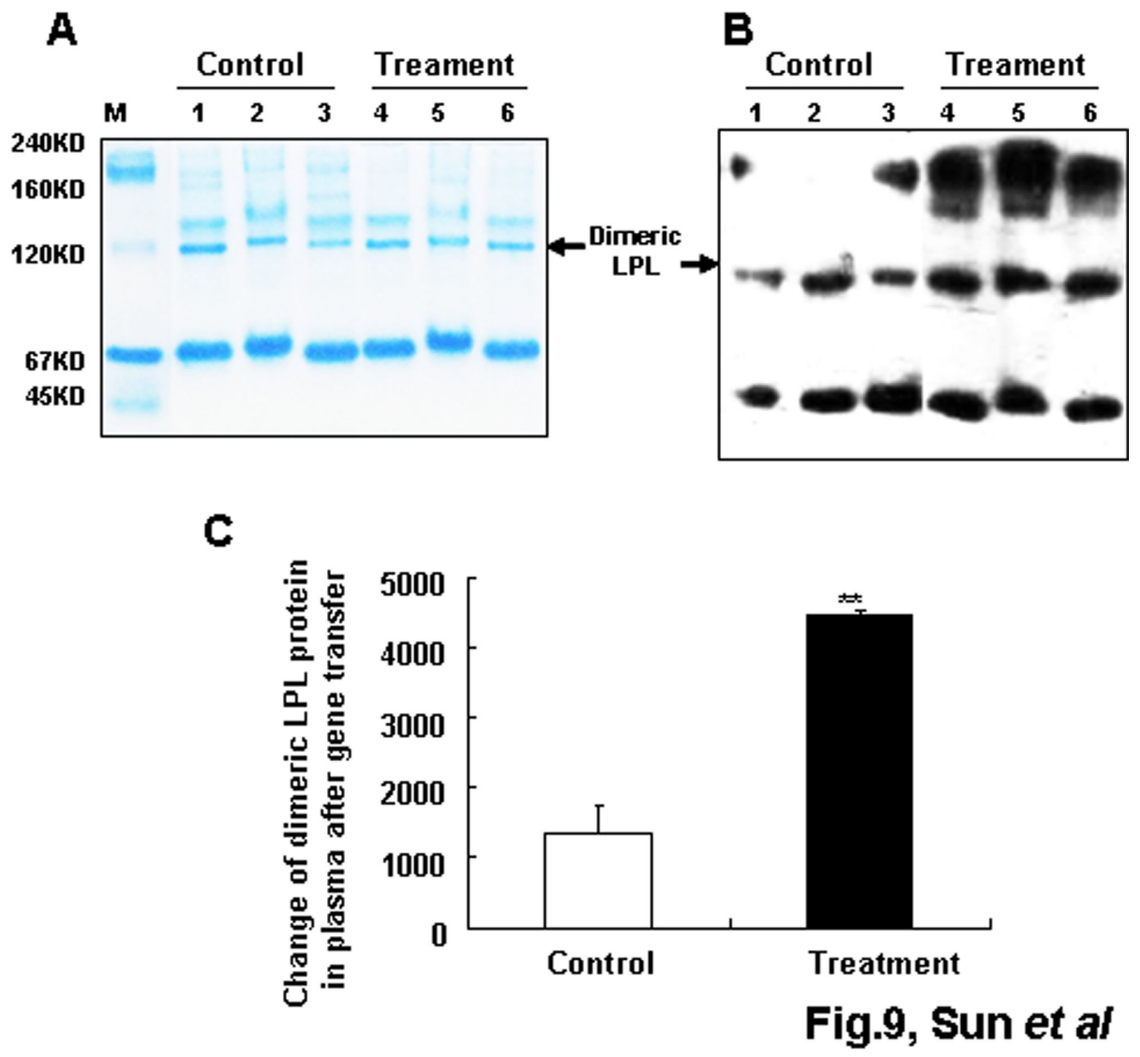
Structure analysis of MLPL in plasma after gene transfer. Coomassie brilliant-stained gel after native PAGE (A) and western blot analysis of plasma MLPL after the gene transfer *in* *vivo* (B and C). Lanes: M, native PAGE protein marker; 1–3, control serum from three mice; 4–6, MLPL gene transferred serum from three mice. Each value represents mean + SEM of three mice. ** indicates significant difference relative to the control at p<0.01.

To ascertain whether Leu452His mutation of LPL affects the formation of bioactive dimer LPL after mutated gene transfer, native PAGE and western blot analysis were performed. The results are shown in Figure 9A to 9C. The plasma MLPL bioactive dimer in the MLPL gene-transferred group was significantly increased compared with that in the control group (p<0.01). These results suggest that the Leu452His mutation of LPL did not affect dimerization of LPL.

## Discussion

In this study, we constructed a pTarget/MLPL vector to express MLPL in mice *in vivo*. Sequence analysis revealed an A◊T substitution at position 1355 of the cDNA. As a result, the histidine residue at the 452^th^ position of the protein was replaced with leucine. Furthermore, this mutation lowered LPL physiological function and produced antagonistic effects as revealed by *in vivo* overexpression of the MLPL gene in mouse liver by HD.

The increase in the mRNA of MLPL in the liver and serum MLPL, and the reduction in LPL activity on days 1 and 7 after HD gene transfer (Figure 2A to 2C) suggest that the overexpressed recombinant MLPL was successfully expressed and secreted into the blood from the liver. Furthermore, MLPL could reach peripheral tissues via blood circulation and could affect the activity of resident LPL of plasma after the gene transfer.

LPL has an important function in TG metabolism. It has been shown that defective LPL can cause hypertriglyceridemia [18–20]. The observed increase in plasma and liver TG on day 7 after the gene transfer (Figures 3B and 4B) is consistent with previous reports [18,19]. By contrast, studies on overexpression of human LPL in transgenic mice have reported decreased plasma TG level after gene transfer [21–23]. Here, we show that an L to H substitution at position 452 of mouse LPL gene abolishes the physiological function of LPL, which further supports our conclusion, Leu452His mutation led to disfunction of LPL.

Monomeric LPL consists of two domains: a large N-terminal domain (amino acid residues 1 to 315) and a small C-terminal domain (amino acid residues 316 to 448) [8]. The N-terminal domain contains the heparin-binding sites and the active site region, including the substrate-binding site and the catalytic center [24]. The C-terminal domain is involved in heparin and substrate binding and is also responsible for the formation and stability of LPL head-to-tail non-covalent homodimer, a configuration necessary for the activity of enzymes [25–28]. Previous studies have shown that LPL mutations affect LPL functions via changes in catalytic activity, dimerization, secretion, and heparin binding [6,8,11]. In our study, the polar amino acid H was changed to the nonpolar amino acid L in the C-terminal of LPL. Thus, this mutation could affect dimerization, heparin binding, and interaction with cofactors of LPL. We tested whether this mutation affected dimerization of LPL by native PAGE and western blot analyses. The results shown in Figure 9A to 9C reveal that the Leu452His mutation of LPL did not affect the dimerization of LPL as expected. The enzymatic activity of LPL requires cofactors such as apoC-II [29], and apoC-II interacts with the last half of the C-terminal domain (residues 389 to 474) to achieve maximal lipolytic activation. In addition, the relative heparin affinity of LPL is determined by the final 60 C-terminal amino acid residues. Thus, dysfunction of LPL with Leu452His mutation observed in our study may result from reduction of substrate binding or interaction between MLPL and cofactors. Future studies should address these possibilities. Nevertheless, the results obtained in this study further support the importance of the C-terminus in LPL enzyme activity.

To investigate whether the MLPL produced in the liver after gene transfer affects energy metabolism in the liver, epidermal white fat, gastrocnemius *muscle*, and *extensor digitorum longus*, expression of genes related to energy metabolism, namely, *HSL*, *ATGL*, *FABP3*, *FABP4*, *UCP2*, and *UCP3*, were investigated. The results presented in Figures 4C, 5A, 6A, and 7A indicate that MLPL produced in the liver after gene transfer can target liver, fat, and muscle tissues, and reduce energy expenditure by competitively targeting LPL substrate in these tissues with endogenous LPL. Furthermore, gene expression in the treatment group on day 1 after the gene transfer was lower compared with that on day 7 after the gene transfer. However, no significant changes in plasma glucose, food intake, body weight and fat tissue were observed, in spite that of the viscera index of liver in the MLPL gene-transfer group significantly increased compared with that in the control group (data not shown). At these points of no change on palsma glucose, food intake, body weight and fat tissue, they were consistent with the specifics of the HD method. The concentration of the final product quickly reached its peak within 24 hours after the gene transfer and was rapidly restored to normal levels on days 2 and 7 after the gene transfer [30,31]. Therefore, the increase in MLPL concentration by HD was rapidly dropped in the latter part of the experiment. Thus, we did not observe remarkable changes in plasma glucose, food intake, body weight and fat.

Notably, *MLPL* gene transfer markedly increased the mRNA level of MLPL+*LPL* in the liver, white fat, and muscles. *MLPL* mRNA was likely produced in the liver after the gene transfer.

During energy metabolism, the AMPK/PGC-1α signaling pathway plays important roles in fat and muscle tissues. In our previous studies, we found that leptin and adiponectin gene transfer by HD could regulate energy metabolism by regulating AMPK/PGC-1α transcription [32,33]. To ascertain whether MLPL can also regulate them as leptin and adiponection, we investigated the expression and protein levels of SIRT1, FOXO3a, AMPK, and PGC-1α in the liver, fat and muscle tissue after the gene transfer. The results presented in Figures 4D, 4E, 4F, 5B, 6B, and 7B to 7D elucidate that MLPL inhibited the energy expenditure of the liver, fat and muscle tissues by reducing the expression levels of AMPK, phospho-AMPK, SIRT1, and PGC-1α at the gene and protein levels.

**Figure 10:**
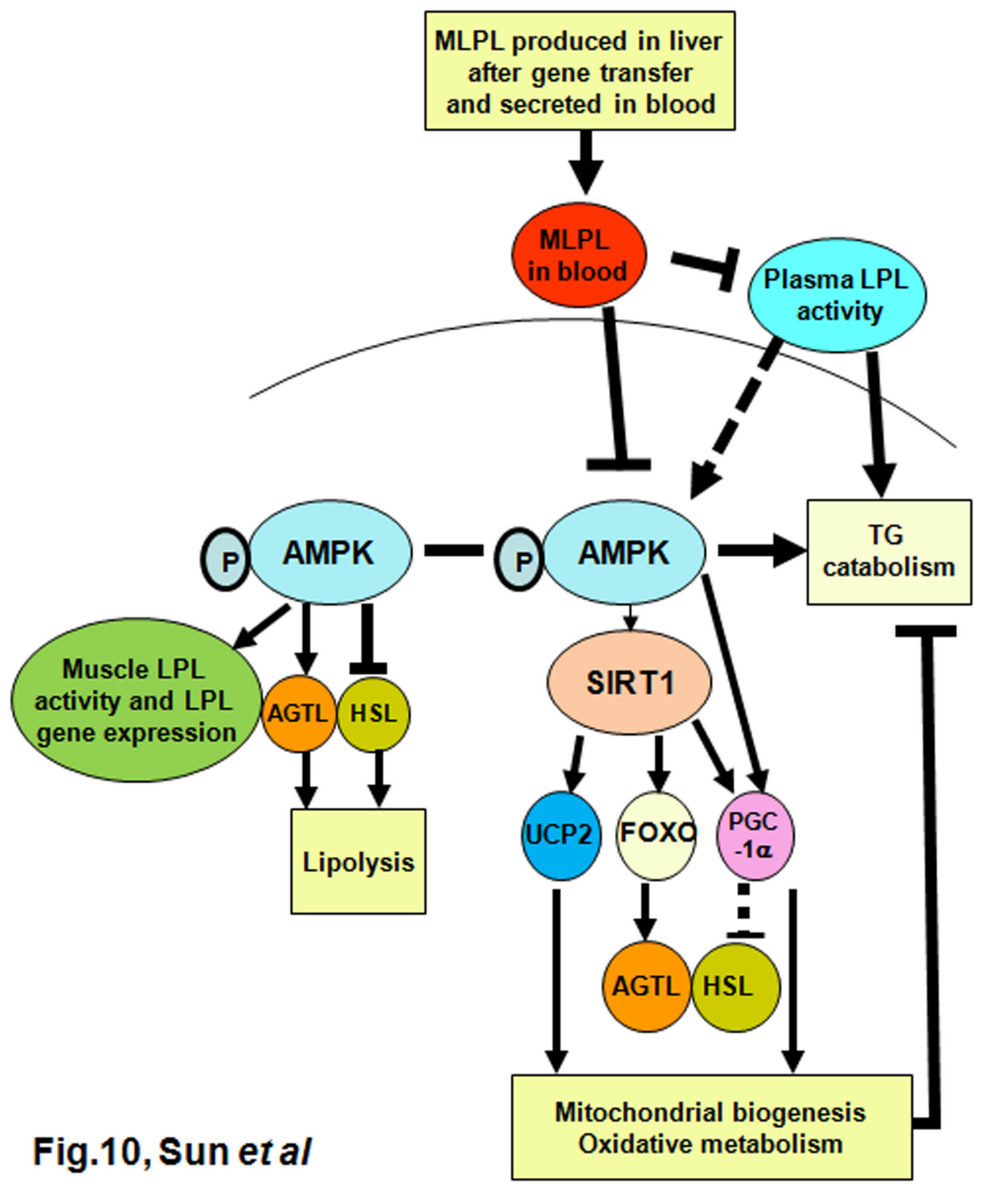
The proposed mechanism of MLPL in regulation of energy metabolism. MLPL regulates energy metabolism via inhibition of the AMPK/SIRT1/PCG1-α signaling pathway.

In summary, this study is the first to report that the Leu452His mutation of mouse MLPL causes LPL dysfunction and that Leu425His MLPL gene transfer can increase the plasma and liver TG contents of mice by antagonizing endogenous LPL. Moreover, *MLPL* gene transfer can decrease energy expenditure in peripheral tissues by inhibiting AMPK, SIRT1, and PGC-1α (Figure 10). In this context, a pathological model may be constructed to study LPL dysfunction-induced diseases, such as type 2 diabetes, coronary disease, and hypertriglyceridemia.
